# Effect of Inhalation of Lavender Essential Oil on Vital Signs in Open Heart Surgery ICU

**Published:** 2017

**Authors:** Armaiti Salamati, Soheyla Mashouf, Faraz Mojab

**Affiliations:** a *Faculty of Nursing and Midwifery, Islamic Azad University (Tehran Medical Branch), Tehran, Iran. *; b *School of Pharmacy, Pharmaceutical Sciences Research Center, Shahid Beheshti University of Medical Sciences, Tehran, Iran.*

**Keywords:** Aromatherapy, Lavender, Vital Sings, Open-heart surgery

## Abstract

This study evaluated the effects of inhalation of Lavender essential oil on vital signs in open heart surgery ICU. The main complaint of patients after open-heart surgery is dysrhythmia, tachycardia, and hypertension due to stress and pain. Due to the side effects of chemical drugs, such as opioids, use of non-invasive methods such as aromatherapy for relieving stress and pain parallel to chemical agents could be an important way to decrease the dose and side effects of analgesics.

In a multicenter, single-blind trial, 40 patients who had open-heart surgery were recruited. Inclusion criteria were full consciousness, lack of hemorrhage, heart rate >60 beats/min, systolic blood pressure > 100 mmHg, and diastolic blood pressure > 60 mmHg, not using beta blockers in the operating room or ICU, no history of addiction to opioids or use of analgesics in regular, spontaneous breathing ability and not receiving synthetic opioids within 2 h before extubation. Ten minutes after extubation, the patients› vital signs [including BP, HR, Central Venous Pressure (CVP), SPO2, and RR] were measured. Then, a cotton swab, which was impregnated with 2 drops of Lavender essential oil 2%, was placed in patients’ oxygen mask and patients breathed for 10 min. Thirty minutes after aromatherapy, the vital signs were measured again. Main objective of this study was the change in vital sign before and after aromatherapy. Statistical significance was accepted for P < 0.05. There was a significant difference in systolic blood pressure (p > 0.001), diastolic blood pressure (p = 0.001), and heart rate (p = 0.03) before and after the intervention using paired t-test. Although, the results did not show any significant difference in respiratory rate (p = 0.1), SpO2 (p = 0.5) and CVP (p = 0.2) before and after inhaling Lavender essential oil. Therefore, the aromatherapy could effectively reduce blood pressure and heart rate in patients admitted to the open heart surgery ICU and can be used as an independent nursing intervention in stabilizing mentioned vital signs. The limitations of our study were sample size and lack of control group. Randomized clinical trials with larger sample size are recommended.

## Introduction

Nowadays, heart surgery is one of the most common surgeries and the development of cardiac surgery and cardiopulmonary bypass techniques has reduced the mortality rates of these surgeries but it has its own side effects (1). The problems in surgery are the neuroendocrine, metabolic, and inflammatory aspects of injury that are part of the overall ‘stress response’ in relation to surgery. Catecholamines are released from the adrenal medulla and norepinephrine spills over from presynaptic nerve terminals in response to hypothalamic stimulation. Marked activation of the sympathetic nervous system results in tachycardia, hypertension and other side effects (2). Whatever the extent of the surgery is greater, equally the physiological changes that occur are greater like heart surgery (3). All heart surgeries make variable levels of pain and postoperative pain for patients, which are not negligible at all (4). Researches have shown that patients that their postoperative pain is poorly controlled may experience heart failure and infection three and five times more than others, respectively (5). Moreover, open-heart surgery pain and stress occur during 24-72 h after surgery (6) and it usually increases by stressful environmental factors and surgical conditions like coughing, movement, and changing position (7). Meanwhile, open-heart surgery pain causes ineffective respiration that will make a delay in leaving the bed, immobility, and stagnation of blood flow and the risk of pulmonary embolism (6). Also, acute pain effects on the immune, respiratory, cardiovascular, gastrointestinal, and endocrine systems. In addition, the postoperative stress and pain actuate the sympathetic system that will cause to increase blood pressure, pulse, heart rate and breathing increases and becomes superficial as well. In fact, each of them increases oxygen demand required by the body, which will cause pressure on the heart muscle and subsequently, the pressure on the heart will increase. All these situations are very dangerous, especially in patients undergoing cardiac surgery (8).

Recent evidences indicated that the pain was not given appropriate and adequate care in more than 75% of open-heart surgery patients and patients had painful experiences of the time in hospital (5). Wang *et al.* also wrote that although the pain of incision was inevitable, it was controllable and in the absence of rapid and appropriate controls, it could become severe and lead to chronic pain (9). Besides, it could cause immobility, reduction of pulmonary ventilation, and consequently delayed recovery, prolonged hospital admission, and increasing the costs (10). Postoperative stress and pain control are one of the major challenges for nurses. They can use pharmacological and non-pharmacological methods (11). Morphine is the most common used pain relievers after heart surgery (7). The opioids have some unwanted side effects that may include nausea and vomiting, dizziness, drowsiness, hypotension, constipation, and respiratory depression (12). It also can increase patient›s tolerance to the drugs (7).

Drugs are not the only way to control stress and pain, although they are the most effective available means for nurses (13). Due to the side effects of chemical drugs and opioids, some procedures with low complications will be used to relieve the stress and pain as a nursing skill (14). Conceivably, not only the use of non-invasive methods can be effective in relieving stress and pain but also the side effects of taking too many analgesics, which in many cases are threatening the health and lives of patients, can decrease. However, the patient suffering from stress and pain can benefit from various methods of complementary medicine. Among these, aromatherapy is a technique of using volatile oils, for psychological and physical health (15). Specifically, efforts to scientifically demonstrate the effects of aromatherapy as a holistic intervention and as a relaxation mediator have been actively pursued in nursing (16). Aromatherapy has been reported to reduce stress (17, 18) as well as increasing sleep quality (19). Previously, the effects of some other herb extracts were exanimate clinically (20-21).

Lavender (*Lavandula officinalis*) from Labiatae family with many therapeutic properties is vastly used in the variety of aromatherapy methods (22). The primary components of lavender oil are linalool (51%) and linalyl acetate (35%). Other components include α-pinene, limonene, 1,8-cineole, cis- and trans-ocimene, 3-octanone, camphor, caryophyllene, terpinen-4-ol, and lavendulyl acetate (23). Applying a few drops of Lavender essential oil to handkerchief and inhaling it is useful for treating insomnia, fatigue, stress, and fear. Warm compress is used in menstrual cramps, stomachache, arthritis, migraines and muscle cramps (22).

It is the first assessment report of the effect of Lavender essential oil on vital signs in Open heart surgery ICU. The researchers studied on the effectiveness of aromatherapy with Lavender essential oil on vital signs of open-heart surgery patients with fewer side effects.

## Methods

This study is a single-blind clinical trial, which has the ethical approval from Ethics Committee of Islamic Azad University of Tehran Medical Branch and registered with code IRCT2013061813711N1 in Iranian registry of clinical trials, and was conducted on 40 patients who had undergone open-heart surgery, cardiac ICU departments in Moheb Hospital and Tehran Heart Center. The Lavender oil was prepared by hydrodistillation from *Lavandula officinalis* (*L. vera*) collected from botanical garden of School of Pharmacy in Jan. 2012, and was identified (by Mr. Kamali-nejad) in Pharmacognosy Lab. in School of Pharmacy, 2% solution was prepared by sesame oil (Saman Co. Iran).. Characteristic subjects of patients under investigation included: full Consciousness and being aware of time and place, not using beta blockers in the operating room or ICU for any reason, extubated and having spontaneous breathing and no history of addiction to opioids or use of analgesics in regular ( codeine, oxycodone, ibuprofen, advil, aspirin and the other non-steroidal anti-inflammatory drugs (NSAIDs). Exclusion criteria were hemorrhage, heart rate <60 beats/min and hypotension (systolic blood pressure < 100 mmHg and diastolic blood pressure ˂ 60 mmHg), receiving analgesics within 2 h before intervention, having asthma, allergies, chronic obstructive pulmonary and other lung diseases and contact dermatitis to aromatic substances. The patients that needed medical intervention during the inhalation, or in case of allergies, respiratory problems and emergencies, were excluded from this research.

**Figure 1:**
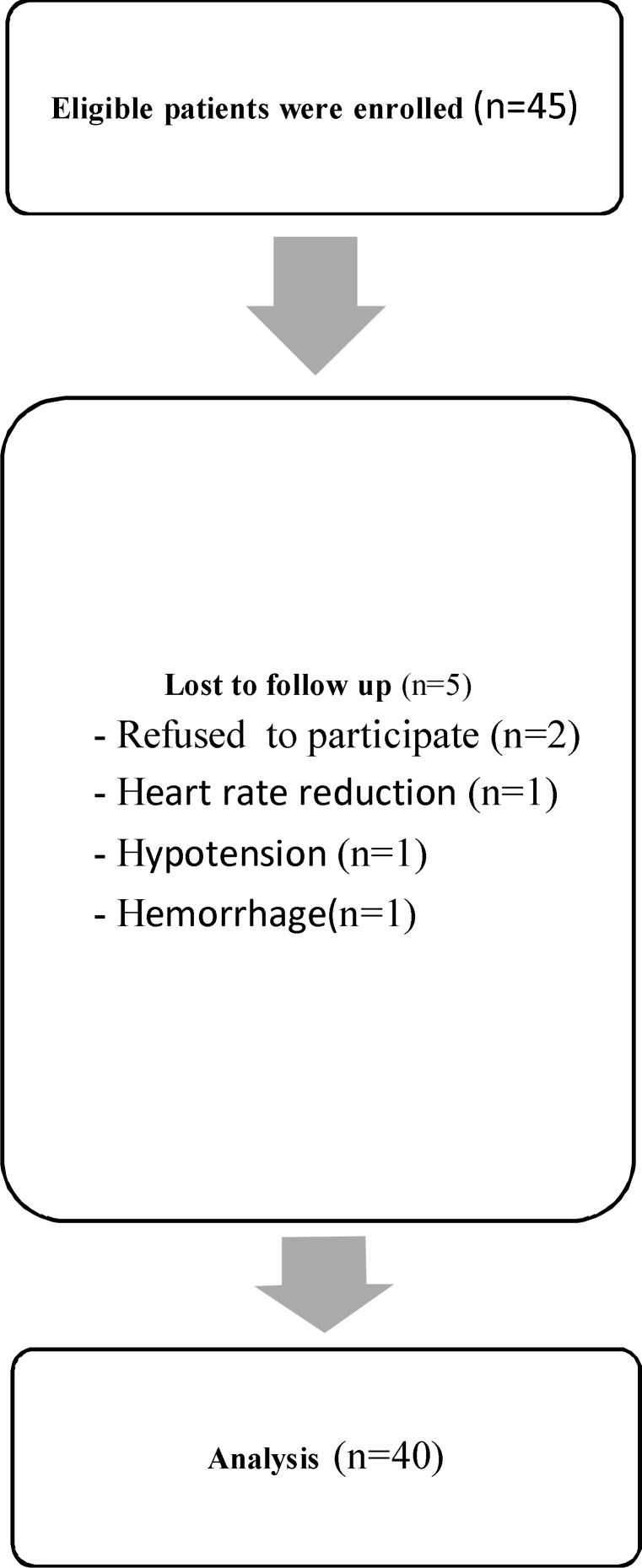
Follow-up of 45 patients.

**Table 1:** Systolic, Diastolic blood pressure and Heart rate average comparison before and after aromatherapy

**Physiological variables**	**Systolic blood pressure average**	**Diastolic blood pressure average**	**Heart rate average**
Before aromatherapy	123.7	73.43	93.12
After aromatherapy	107.3	66.06	85.20

**Table 2:** Respiratory rate, SpO2 rate and Central venous pressure average comparison before and after aromatherapy

**Physiological variables**	**Respiratory rate average**	**SpO2 rate average**	**CVP average**
Before aromatherapy	20.48	95.60	10.20
After aromatherapy	20.32	94.99	10.48

A two-part questionnaire was used in this study for the data collection. The first part included demographic characteristics subjects under investigation (age, gender, income, education, marital status, place of residence, etc.) and the second part was used a Japanese analog barometer in order to measure the blood pressure, The respiratory rate, pulse, SpO2, and central venous pressure (CVP).

All patients, who were enrolled to the study after Filling out the informed consent form, received a lavender oil patch test, preoperatively. In order to reduce inhalation and skin absorption of the lavender, One drop of lavender oil was dropped to their ankle, the test patch was immediately covered with an occlusive dressing and removed after a two-minute exposure. Then, on the day of surgery, ten minutes after extubation, the vital signs were measured by a nurse. Then a cotton swab, which was impregnated with 2 drops of Lavender 2%, was placed in patients’ oxygen mask, and patients breathed for 10 min. After thirty minutes, the vital signs were measured again (Figure 1). SPSS 18.0 (SPSS Inc, Il, USA) was used for statistical analysis. The patient’s vital signs before and after aromatherapy were analyzed by paired t-tests and chi-square and Fishers’ exact were used to investigate the relationship between demographic characteristics and vital signs. Statistical significance was accepted for P < 0.05.

## Results

Demographic data from participants showed that 57.5% of samples were males, patients’ age was in the range of 18-65 and majority of samples (47.5%) were 50 to 59 years and average age was 50 years old (SD = 15.16). 37.5% Of patients had hypertension, 30% had hyperlipidemia and 20% had diabetes. 57.5% had an experience of previous surgery and only 10% had an experience of aromatherapy. None of the subjects had previously experience of using Lavender essential oil.

The statistical paired t-test before and after aromatherapy showed that there were significant differences in systolic blood pressure (p > 0.001), diastolic blood pressure (p = 0.001), and heart rate (p = 0.03). Although, the results did not show any significant differences in respiratory rate (p = 0.1), SpO2 rate (p = 0.5) and Central venous pressure (CVP) (p = 0.2) before and after inhaling Lavender essential oil. 

Therefore, the aromatherapy effectively reduced blood pressure and heart rate in patients admitted to the Open heart surgery ICU and can be used as an independent nursing intervention for reducing BP and HR. (Table 1 and 2).

Chi-square statistical test is used to investigate the relationship between demographic characteristics and vital signs but there were not significant effects between demographic variables and physiological variables. 

## Discussion

The results of our investigation showed that there were significant differences in systolic blood pressure, diastolic blood pressure, and heart rate before and after aromatherapy, but these differences in respiratory rate, SpO2 rate and central venous pressure were not statistically significant. 

In 2014, Lytle *et al*., studied on the «Effect of lavender aromatherapy on vital signs and perceived quality of sleep in the Intermediate Care Unit (24). In this study blood pressure was significantly lower in the treatment group than in the control group and the results are similar. In 2006, Hwang JH, has done another study with the title «The effects of the inhalation method using essential oils on blood pressure and stress responses of clients with essential hypertension” the inhalation method of blending oils with lavender, ylangylang, and bergamot was used. The results suggest that the inhalation method using essential oils can be considered an effective nursing intervention that reduces blood pressure, psychological stress responses, and serum cortisol levels of clients with essential hypertension (18).

But in 2013, Cho *et al*., studied on the «Effects of aromatherapy on the anxiety, vital signs, and sleep quality of percutaneous coronary intervention patients in intensive care units» their results showed systolic and diastolic blood pressure did not have any significant differences and their results were quite different from ours, although outcome measures patients› state anxiety, sleeping quality significantly low anxiety and improving sleep quality compared with conventional nursing intervention (25).

Essential oils have both physiological and psychological effects (26). When lavender oil is inhaled for 10 min, there is an increase in blood flow rate and a decrease in galvanic skin conduction and systolic blood pressure (indicating a reduction in sympathetic nerve activity) (27). Human studies with lavender have demonstrated a significant relaxation effect and reduced anxiety, but no direct antinociception (28, 29).
